# Correction: Acquired resistance of *Stenotrophomonas maltophilia* to antimicrobials induced by herbicide paraquat dichloride

**DOI:** 10.1371/journal.pone.0346751

**Published:** 2026-04-08

**Authors:** The PLOS One Staff

## Notice of Republication

This article was republished on March 25, 2026, to correct an error in the author list. Paiboon Vattanaviboon was erroneously displayed as Paiboon Vattanavibooon. The publisher apologizes for the error. Please download this article again to view the correct version. The originally published, uncorrected article and the republished, corrected articles are provided here for reference.

## Supporting information

S1 File Originally published, uncorrected article. (PDF)

S2 File Republished, corrected article. (PDF)
