# Supplementary material for: Acquired resistance of Stenotrophomonas maltophilia to antimicrobials induced by herbicide paraquat dichloride
Source: PLoS One. 2024 Aug 28;19(8):e0309525. doi: 10.1371/journal.pone.0309525 (PMC11356428; doi:10.1371/journal.pone.0309525)
Supplement: S1 Fig — Real-time RT-PCR was used to assess the expression levels of potential antibiotic resistance genes in both wild-type and mutant strains of S. maltophilia K279a. The melting curves from a representative real-time RT-PCR experiment for all amplified genes are shown. (PPTX) [file pone.0309525.s001.pptx]

## Slide 1
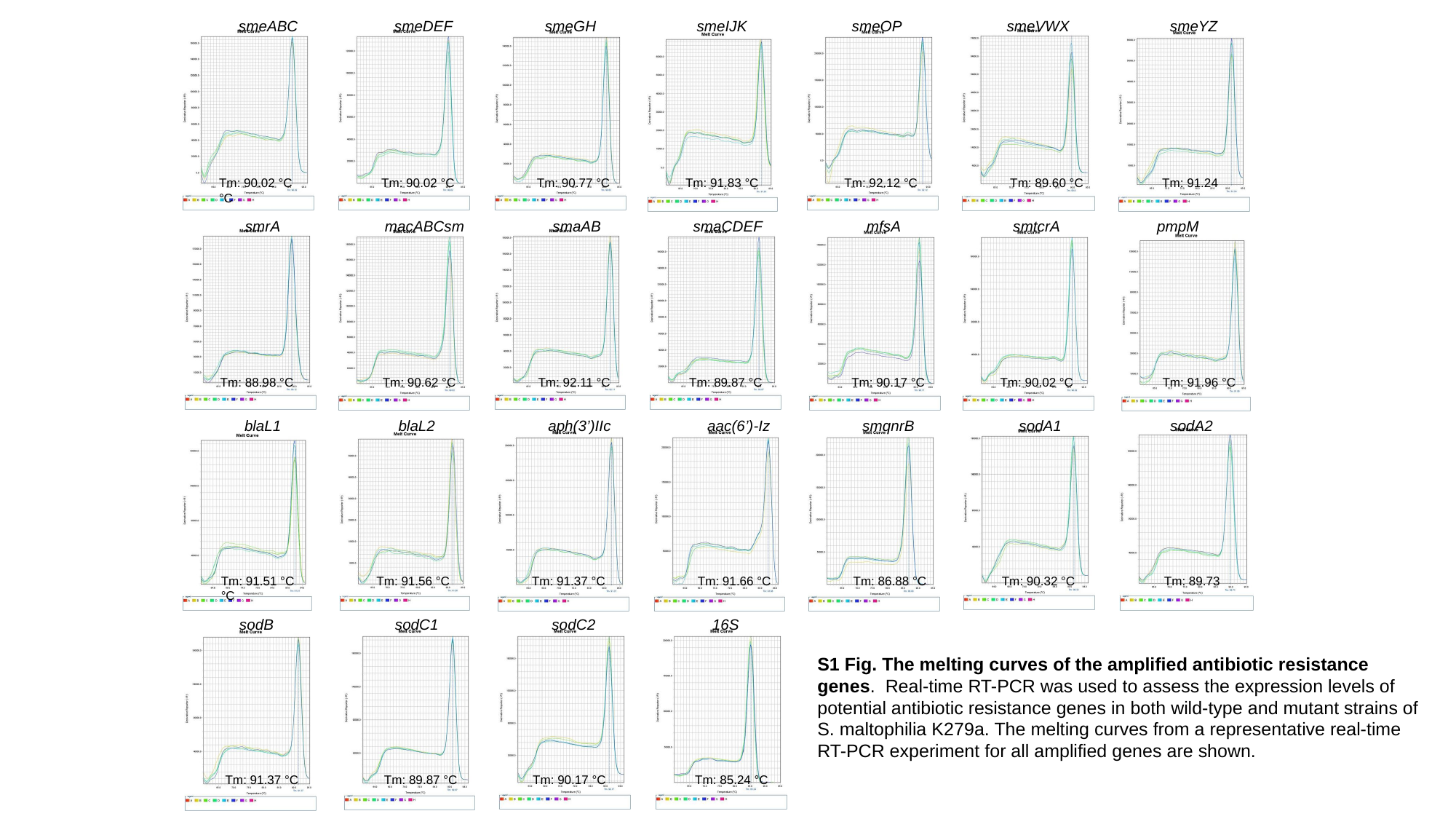

smeABC smeDEF smeGH smeIJK smeOP smeVWX smeYZ
Tm: 90.02 °C Tm: 90.02 °C Tm: 90.77 °C Tm: 91.83 °C Tm: 92.12 °C Tm: 89.60 °C Tm: 91.24 °C
 smrA macABCsm smaAB smaCDEF mfsA smtcrA pmpM
Tm: 88.98 °C Tm: 90.62 °C Tm: 92.11 °C Tm: 89.87 °C Tm: 90.17 °C Tm: 90.02 °C Tm: 91.96 °C
blaL1 blaL2 aph(3’)IIc aac(6’)-Iz smqnrB sodA1 sodA2
Tm: 91.51 °C Tm: 91.56 °C Tm: 91.37 °C Tm: 91.66 °C Tm: 86.88 °C Tm: 90.32 °C Tm: 89.73 °C
sodB sodC1 sodC2 16S
Tm: 91.37 °C Tm: 89.87 °C Tm: 90.17 °C Tm: 85.24 °C
S1 Fig. The melting curves of the amplified antibiotic resistance genes. Real-time RT-PCR was used to assess the expression levels of potential antibiotic resistance genes in both wild-type and mutant strains of S. maltophilia K279a. The melting curves from a representative real-time RT-PCR experiment for all amplified genes are shown.
