# Supplementary material for: Acquired resistance of Stenotrophomonas maltophilia to antimicrobials induced by herbicide paraquat dichloride
Source: PLoS One. 2024 Aug 28;19(8):e0309525. doi: 10.1371/journal.pone.0309525 (PMC11356428; doi:10.1371/journal.pone.0309525)
Supplement: S2 Fig — Pairwise alignment between the acnA gene (smlt3608) and the insert in pAcnA was conducted using the ClustalW algorithm (https://www.genome.jp/tools-bin/clustalw). An asterisk (*) indicates identical sequences. The start (ATG) and stop (TGA) codons are in bold font. (PPTX) [file pone.0309525.s002.pptx]

## Slide 1
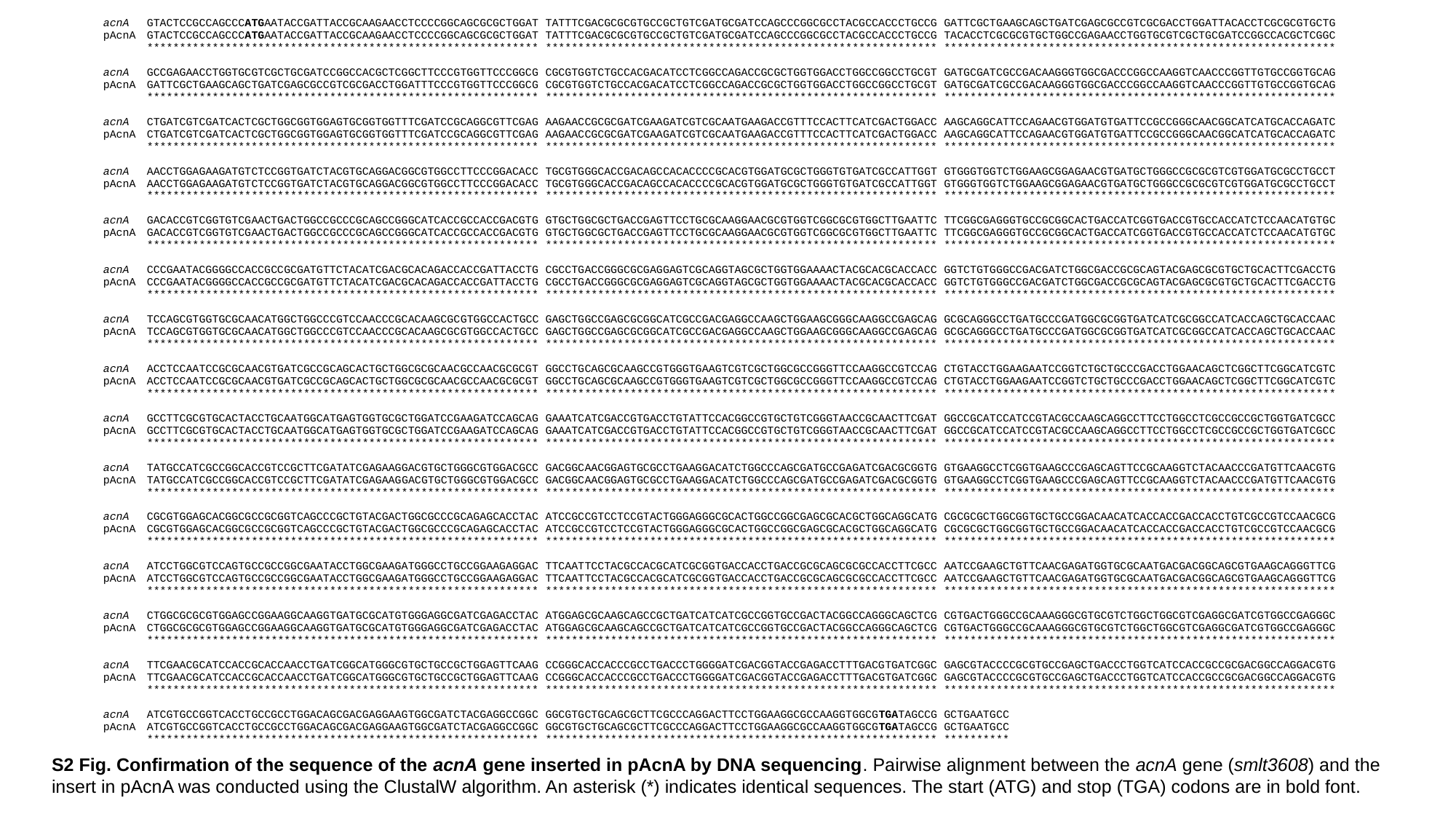

S2 Fig. Confirmation of the sequence of the acnA gene inserted in pAcnA by DNA sequencing. Pairwise alignment between the acnA gene (smlt3608) and the insert in pAcnA was conducted using the ClustalW algorithm. An asterisk (*) indicates identical sequences. The start (ATG) and stop (TGA) codons are in bold font.
