# Supplementary material for: Correction: Acquired resistance of Stenotrophomonas maltophilia to antimicrobials induced by herbicide paraquat dichloride
Source: PLoS One. 2026 Apr 8;21(4):e0346751. doi: 10.1371/journal.pone.0346751 (PMC13061165; doi:10.1371/journal.pone.0346751)
Supplement: S1 File — (PDF) [file pone.0346751.s001.pdf]

PLOS ONE

RESEARCH ARTICLE

Acquired resistance of *Stenotrophomonas maltophilia* to antimicrobials induced by herbicide paraquat dichloride

Veerakit Vanitshavit[1☯], Nisanart Charoenlap[1,2☯], Ratiboot Sallabhan[1], Wirongrong Whangsuk[1], Kisana Bhinija[1], Punyawee Dulyayangkul[1], Skorn Mongkolsuk[1,2], Paiboon Vattanaviboon[1,2,3]*

1 Laboratory of Biotechnology, Chulabhorn Research Institute, Bangkok, Thailand, **2** Center of Excellence on Environmental Health and Toxicology (EHT), OPS, MHESI, Bangkok, Thailands, **3** Program in Applied Biological Science: Environmental Health, Chulabhorn Graduate Institute, Bangkok, Thailand

☯ These authors contributed equally to this work.
* paiboon@cri.or.th

Check for updates

OPEN ACCESS

Citation: Vanitshavit V, Charoenlap N, Sallabhan R, Whangsuk W, Bhinija K, Dulyayangkul P, et al. (2024) Acquired resistance of *Stenotrophomonas maltophilia* to antimicrobials induced by herbicide paraquat dichloride. PLoS ONE 19(8): e0309525. https://doi.org/10.1371/journal.pone.0309525

Editor: Seyed Mostafa Hosseini, Hamadan University of Medical Sciences, ISLAMIC REPUBLIC OF IRAN

Received: February 14, 2024

Accepted: August 14, 2024

Published: August 28, 2024

Copyright: © 2024 Vanitshavit et al. This is an open access article distributed under the terms of the Creative Commons Attribution License, which permits unrestricted use, distribution, and reproduction in any medium, provided the original author and source are credited.

Data Availability Statement: All relevant data are within the manuscript and its Supporting Information files.

Funding: This work was supported by Thailand Science Research and Innovation (TSRI), Chulabhorn Research Institute (https://www.cri.or.th) Grant number 48293/4691984 to NC, PV and SM. The funder play no role in the study design, data collection and analysis, decision to publish, or preparation of the manuscript.

Abstract

Stenotrophomonas maltophilia, a ubiquitous environmental bacterium, is an important cause of nosocomial infections. Although banned in some countries, paraquat (PQ) is commonly used to control weeds. In this study, we investigated the effects of increasing concentrations of PQ on *S. maltophilia* and its antimicrobial resistance. The sequential exposure of *S. maltophilia* K279a to increasing concentrations of PQ induces the formation of strains with increased resistance to PQ. Among the 400 PQ-resistant isolates tested, 70 clones were resistant to 16 μg/ml ciprofloxacin (CIP), and around 18% of the PQ/CIP-resistant isolates showed increased resistance to all the tested antimicrobials including, the aminoglycosides, quinolones, cephalosporin, chloramphenicol, and co-trimoxazole. The results of the expression analysis of the antimicrobial resistance genes in the five selected PQ/CIP-resistant isolates demonstrated the high expression of genes encoding efflux pumps (*smeYZ*, *smaAB*, *smaCDEF*, *smeDEF*, *smeVWX*, and *smtcrA*) and the enzymes *aph(3')-IIc*, *blaL1*, and *blaL2*. However, expression of the genes known for PQ resistance (i.e., *mfsA* and *sod*) were not altered relative to the wild-type levels. Whole genome sequence analysis identified gene mutations that could account for the antimicrobial resistance, namely, *smeT* (TetR family regulatory protein), *rplA* (ribosomal protein L1), and *acnA* (aconitase A). Ectopic expression of wild-type AcnA partially complemented the fluoroquinolone-resistant phenotype of the mutant with mutated *acnA*, which suggests the role of aconitase A in antimicrobial susceptibility. Exposure of *S. maltophilia* to PQ thus induces the development of strains that increase resistance to multiple antimicrobials.

Introduction

Stenotrophomonas maltophilia is a gram-negative aerobic bacterium, which is recognized as one of the major causes of hospital-acquired infections (HAIs) worldwide. The incidence of

Competing interests: The authors have declared that no competing interests exist.

community-acquired *S. maltophilia* infections is also increasing [1]. HAIs are generally associated with high mortality and increased morbidity, length of hospital stay, and medical treatment costs. Resistance to multiple antimicrobial agents poses a major challenge to the management of nosocomial infections caused by *S. maltophilia*. This bacterium has developed an array of resistance mechanisms, including the production of antibiotic-modifying and -degrading enzymes and multidrug efflux transporters, a reduction in outer membrane permeability, and the modification of antibiotic targets, which make *S. maltophilia* intrinsically resistant to multiple antimicrobials [2,3]. The resistance of *S. maltophilia* to antimicrobial agents can be acquired by the acquisition of resistance genes through horizontal gene transfers from microorganisms that reside in the same habitats as well as the mutations of genes associated with antimicrobial resistance [4].

Paraquat dichloride (1,1′-dimethyl-4-4′-bipyridinium dichloride, PQ), also known as methyl viologen, is an active ingredient of non-selective herbicides. PQ is commonly used to control weeds and grasses in agriculture, even though its use has been banned or restricted in more than 67 countries [5]. PQ exerts its herbicidal activity by interfering with photosynthesis pathways. It accepts a free electron from the ferredoxin photosystem I to form a PQ radical cation ($PQ^{\cdot +}$), which immediately reacts with oxygen to form highly reactive superoxide anions while concomitantly generating the parent PQ, which is recycled to continuously produce superoxide anions [6]. Extensive cellular damage from excessive reactive oxygen species (ROS) causes cell membrane disruption, which leads to leaf wilting, chlorosis, and eventually, desiccation [7]. As a redox cycling substance, PQ is potentially hazardous to non-target organisms, including humans, animals, and microorganisms.

PQ has shown to be very immobile and highly persistent in soil. It does not hydrolyze or photodegrade in aqueous solutions and is resistant to microbial degradation [8]. As *S. maltophilia* is an ubiquitous environmental bacterium, it may be exposed to PQ contamination in soil and aquatic environments. Little is known about the responses of *S. maltophilia* against PQ. It has previously been demonstrated that the expression of *mfsA* encodes an efflux transporter for PQ, and fluoroquinolone antibiotics are controlled by SoxR, a superoxide-sensing transcriptional regulator. Without treatment with PQ and other superoxide generators, SoxR slightly represses *mfsA* expression. Upon exposure to PQ, oxidized SoxR triggers the expression of *mfsA*, thereby inducing cross-protection to PQ [9]. High expression of *mfsA* also increases resistance to fluoroquinolones [10]. PQ exerts its toxicity to living cells primarily through the generation of superoxide anions. The functions of three superoxide dismutase (SOD) enzymes, namely, two manganese-containing SODs (SodA1 and SodA2) and an iron-containing SOD (SodB), in *S. maltophilia* have been characterized [11]. The *S. maltophilia* K279a genome contains two more Cu/Zn SODs (Smlt0160 and Smlt0161) [2]. The expression of *sodA1* is presumably regulated by SoxR because the menadione (superoxide generator)-mediated upregulation of *sodA1* is abolished in the *soxR* mutant [11]. It is thus likely that SoxR modulates the cross talk between the oxidative stress response and protection against antibiotics. We show herein that the exposure of *S. maltophilia* K279a to PQ could induce the generation of mutant strains that showed increased resistance to ciprofloxacin (CIP) as well as other quinolones and several classes of antimicrobial agents.

Materials and methods

Bacterial growth conditions and selection of PQ and CIP resistance

The clinical isolate *S. maltophilia* K279a [2] was used to select PQ-resistant strains by applying a serial passage method as previously described [12], with some modifications to the culturing conditions. A single colony of K279a was inoculated into a lysogenic broth (LB) and incubated

at 35˚C for 24 h with shaking at 150 rpm. After incubation, bacterial cells were inoculated into an LB that had been supplemented with 500 μg/ml PQ (MIC of 1,024 μg/ml [13]) at a starting OD_{600} of 0.2 and the culture was incubated for 24 h under the same growth conditions. The subculturing was repeated 7 consecutive times, starting with an initial OD_{600} of 0.2 in fresh LB medium supplemented with increasing concentrations of PQ (750, 1,000, 1,250, 1,500, 2,000, 2,500, and 3,000 μg/ml). The culture was then spread onto the LB plates containing 3,000 μg/ml PQ, and 400 isolated colonies were tested for their ability to grow on Mueller–Hinton agar plates supplemented with 16 μg/ml CIP.

Antimicrobial susceptibility testing

The antimicrobial susceptibility of the *S. maltophilia* strains was determined using the standard Kirby–Bauer disk diffusion method [14]. The following antibiotic disks (purchased from Oxoid, Hampshire, U.K.), were used: ciprofloxacin (CIP 5 μg), levofloxacin (LEV 5 μg), moxifloxacin (MXF 5 μg), nalidixic acid (NA 30 μg), amikacin (AK 30 μg), gentamicin (GM 10 μg), netilmicin (NET 30 μg), ceftazidime (CAZ 30 μg), cefoperazone (CFP 30 μg), meropenem (MEM 10 μg), chloramphenicol (C 30 μg), and co-trimoxazole (SXT 25 μg), polymyxin B (PB 300 μg), azithromycin (AZM 15 μg), polymyxin B (PB 300 U), amoxycillin/clavulanic acid (AMC 30 μg). Quality assurance of antimicrobial susceptibility testing using disk diffusion assays was routinely performed with *Escherichia coli* ATCC 25922, following the Clinical & Laboratory Standards Institute (CLSI) guidelines [15].

A disk diffusion assay was also used to determine the levels of resistance against oxidants. The disks were prepared by soaking the blank disks in 10 μl 0.5 M PQ, 2.0 M menadione, and 0.5 M phenazine and placing them on a lawn of cells instead of antimicrobial disks. The data were shown as the mean of the three replications ± standard deviation (SD).

Whole genome sequencing and mutation analysis

Whole genome sequencing was performed by Porcinotec (Bangkok, Thailand). The genomic DNA of the *S. maltophilia* strains was isolated using a GF-1 Bacterial DNA Extraction Kit (Vivantis, Malaysia) in accordance with the manufacturer's protocols. The quality of the extracted DNA was determined using a DeNovix QFX fluorometer (Wilmington, DE)

The library preparation of the genomic DNA was performed using a QIAseq FX DNA Library Preparation Kit (Qiagen, Hilden, Germany). The DNA fragments were labeled with different sequencing adaptors. The DNA libraries were checked on QIAxcel Advanced System (Qiagen, Hilden, Germany) and quantified using the DeNovix QFX fluorometer. The DNA libraries were sequenced using V2 chemistry with 2⊆250 bp reads on the Illumina MiSeq platform (Illumina, San Diego, CA). The output reads were processed using Trimmomatic version 0.39 [16], with the parameters set to LEAD:28, TRAILING:28, SLIDING WINDOW:4:15, and MINLEN:36 to remove the adapters and filter the low-quality reads with a quality score less than Q30. FastQC version 0.12.1 was used to assess the quality of the trimmed sequencing reads, which the quality score had to be equal to or greater than Q30 [17].

Snippy version 4.6.0 [18] was used to identify the single nucleotide polymorphisms (SNPs) between the haploid reference genome (*S. maltophilia* K279a; accession no: NC_010943.1) and the sequencing reads. The assembled genomic sequences of *S. maltophilia* K279a variants have been deposited in the NCBI databases under the BioProject (accession number PRJNA1072246).

Real-time reverse transcription PCR

The expression levels of the antibiotic resistance genes were determined using real-time reverse transcription PCR (qRT-PCR), as previously described [19]. The list of genes and primers used are shown in Table 1. The exponentially growing cultures of the *S. maltophilia* strains in the LB medium were harvested for total RNA extraction. The reverse transcription was carried out using 1 μg of DNase I-treated total RNA, a RevertAid Reverse Transcriptase (Thermo Fisher Scientific, Waltham, MA), and random hexamers in line with the manufacturer's recommendations. The cDNA (10 ng) was then used as the DNA templates for amplification of the antibiotic resistance genes using the SYBR Select Master Mix for CFX (Thermo Fisher Scientific) and specific primers (Table 1). The PCRs were run on a StepOnePlus Real-Time PCR System (Thermo Fisher Scientific) for 40 cycles of denaturation at 95˚C for 30 s and annealing at 60˚C for 15 s, with an extension of 30 s at 72˚C. The 16S rRNA, which was amplified with the primers BT2781 and BT2782 (Table 1), was used as the normalized gene. Following PCR, a melting curve analysis was conducted to verify the specificity of the amplified DNA sequences. The melting curves from a representative experiment are displayed in S1 Fig. Then, the results were analyzed based on the normalized gene expression and the cycle threshold method ($2^{-\Delta\Delta C(t)}$) and expressed as fold changes of the expression relative to the level in *S. maltophilia* wild-type K279a. The experiments were repeated independently three times, and the means ± SDs are provided. All expression data are presented in S1 Table.

Cloning of *acnA*

The putative *acnA* gene (*smlt3608*) encoding aconitase A was PCR amplified from *S. maltophilia* K279a genomic DNA with the primers BT8277 and BT8278 using Phusion High-Fidelity DNA Polymerase (Thermo Fisher Scientific). A 2,650 bp amplicon was cloned into pBBR1MCS-3 [20], an *E. coli*/*S. maltophilia* shuttle plasmid vector, cut with *Sma*I, which yielded pAcnA. The recombinant plasmid was verified via DNA sequencing and no mutations were observed (S2 Fig). The pAcnA plasmid was introduced into the KPQC13 mutant using electroporation, as previously described [9].

Statistical analysis

The statistical analyses were performed using SPSS version 23.00 (SPSS Inc., Chicago, IL). One-way ANOVA followed by Dunnett's post-hoc test was used to compare the data pertaining to the *S. maltophilia* variant strains relative to K279a wild-type. A p-value <0.05 was considered statistically significant.

Results

Selection of PQ/CIP-resistant mutants

After the serial passage of the *S. maltophilia* K279a culture in the presence of increasing concentrations of PQ, bacterial culture was spread onto LB plates containing 3,000 μg/ml PQ. Four hundred isolated colonies tolerant to 3,000 μg/ml PQ were then randomly selected and tested for their ability to grow on plates containing 16 μg/ml CIP (the MIC of K279a for CIP was 2 μg/ml). Among the tested isolates, 70 PQ-resistant isolates (70/400, 17.5%) could resist the CIP at 16 μg/ml. The antibiotic susceptibility profiles for representative antibiotics from various classes, including those recommended by the CLSI [15] for the treatment of *S. maltophilia* infections (i.e., quinolones [CIP, levofloxacin, and moxifloxacin], aminoglycosides [amikacin and gentamicin], cephalosporin [ceftazidime], penicillin [amoxicillin/ clavulanic acid], sulfonamide [co-trimoxazole], and chloramphenicol) were determined for all 70 PQ/CIP-

Table 1. List of primers used in this study.

Primer	Sequence (5′→ 3′)	Gene	Product
BT2781	GCCCGCACAAGCGGTGGAG	Forward primer for *16S rRNA*	rRNA
BT2782	ACGTCATCCCCACCTTCC	Reverse primer for *16S rRNA*	rRNA
BT8277	GTACTCCGCCAGCCCATGAAT	Forward primer for *acnA*	AcnA
BT8278	GGCATTCAGCCGGCTATCA	Reverse primer for *acnA*	AcnA
BT8127	CCCGCATCAACCTCGACTAC	Forward primer for *smeABC*	RND
BT8128	CAGCACCTTTACCTGTGCCT	Reverse primer for *smeABC*	RND
BT8129	CAACGTCACCCTCGGCTATG	Forward primer for *smeDEF*	RND
BT8130	CGACGCTCACTTCAGAGAACT	Reverse primer for *smeDEF*	RND
BT8133	CCGATCCACGTCCTGTTCAA	Forward primer for *smeIJK*	RND
BT8134	GTAGACGTACTCGCCATCCG	Reverse primer for *smeIJK*	RND
BT8137	GAACTGGACGTGGCTGACTTC	Forward primer for *smeOP*	RND
BT8138	CAGGTCGATCAGCACTTCGC	Reverse primer for *smeOP*	RND
BT8139	GGAGTTCACCAAGGTGCGT	Forward primer for *smeVWX*	RND
BT8140	GAAGTCGACCTTGCCCGAAT	Reverse primer for *smeVWX*	RND
BT8141	CCAGTGCCGAGTACGAACAG	Forward primer for *smeYZ*	RND
BT8142	CGCGCATCGACATTGATACC	Reverse primer for *smeYZ*	RND
BT7111	TCTGGTACGGATTGGCCTGC	Forward primer for *mfsA*	MFS
BT7112	CCGACCATCGAAGGCACCAC	Reverse primer for *mfsA*	MFS
BT8255	CGGGTGATGATGTCTGGCTT	Forward primer for *smtcrA*	MFS
BT8256	TGAAGGTCACATAGCCGACG	Reverse primer for *smtcrA*	MFS
BT8149	ATCGGCGTGATCGGATTCAT	Forward primer for *mdtD*	MFS
BT8150	CCGATGCGCGAGAAAAGATT	Reverse primer for *mdtD*	MFS
BT8189	GATCCAGGAACTGGCGGTC	Forward primer for *smrA*	ABC
BT8190	CTGGGCGATGGTGGTGATG	Reverse primer for *smrA*	ABC
BT8197	TTGCCGAAGTGGATTCGCAG	Forward primer for *smaAB*	ABC
BT8198	GTGAGACGATGCGGGTGTAG	Reverse primer for *smaAB*	ABC
BT8193	CCGAAGCACAGCTGAAAACTG	Forward primer for *macABCsm*	ABC
BT8194	GGTACTTGCGGTCGGGGTC	Reverse primer for *macABCsm*	ABC
BT9036	TCCGATTCCAGTCCCTCGAT	Forward primer for *smaCDEF*	ABC
BT9037	CGTATCCAGCCCATCGAACT	Reverse primer for *smaCDEF*	ABC
BT8199	TATGCGTTCGCCTTCCTCAC	Forward primer for *pmpM*	MATE
BT8200	GCACCAGCGCTTTCAGGATG	Reverse primer for *pmpM*	MATE
BT8213	GGTCACCTGCTGGACAACAT	Forward primer for *blaL1*	ENZ
BT8214	CACTTCGCCGTCCATGATGA	Reverse primer for *blaL1*	ENZ
BT8215	GGCATTGCTGGACAGGCG	Forward primer for *blaL2*	ENZ
BT8216	GCCCTTGGCAAAGCTGTTCA	Reverse primer for *blaL2*	ENZ
BT8225	TAATTGCCACCGCCGAAGAA	Forward primer for *aph(3')IIc*	ENZ
BT8226	AGTCATCGGCATCCACCAACC	Reverse primer for *aph(3')IIc*	ENZ
BT8231	GACGGTTGGTTTCGCTGAAG	Forward primer for *aac(6')Iz*	ENZ
BT8232	GCGGAAATAGACGACCCGTT	Reverse primer for *aac(6')Iz*	ENZ
BT6866	TCAATGGCGCCACGCTGAAG	Forward primer for *smqnrB*	ARP
BT6867	TCCAGCGTTACCCGCGAGAA	Reverse primer for *smqnrB*	ARP
BT4057	GCCACGCCAACCACAGCC	Forward primer for *sodA1*	SOD
BT4058	AGGATCGGGGTGTTGCCC	Reverse primer for *sodA1*	SOD
BT4610	CAAGAAGCTCAAGTCGCT	Forward primer for *sodA2*	SOD
BT4611	TCGAGAGCACCGGCAACC	Reverse primer for *sodA2*	SOD
BT4608	AAGAGATCGTCCGCCAGG	Forward primer for *sodB*	SOD

(*Continued*)

Table 1. (Continued)

Primer	Sequence (5′→ 3′)	Gene	Product
BT4609	GGTGAAGTAACGCGACTG	Reverse primer for *sodB*	SOD
BT4604	AGACCGAACCTACTCCGC	Forward primer for *sodC1*	SOD
BT4605	CTTCAATCCCGGTGGCGC	Reverse primer for *sodC1*	SOD
BT4606	CGAACCGGCTGCAACGCC	Forward primer for *sodC2*	SOD
BT4607	CACGCGAAGGGCGACTGC	Reverse primer for *sodC2*	SOD

https://doi.org/10.1371/journal.pone.0309525.t001

resistant isolates using a Kirby–Bauer disk diffusion assay. An isolate that showed a smaller inhibition zone diameter (≥5 mm) for each antibiotic than the K279a wild-type was considered to have significantly increased resistance, as demonstrated in a previous study [21]. All 70 isolates showed increased resistance to CIP, levofloxacin, and chloramphenicol, as illustrated in Fig 1. In the case of levofloxacin, all the isolates were categorized as "resistant" as per the CLSI guidelines (inhibition zone diameter ≤13 mm) [15]. Moreover, we found that 85% of the isolates showed increased resistance to amikacin, gentamicin, and amoxicillin/clavulanic acid, while 67% showed increased resistance to ceftazidime. About half the isolates displayed increased resistance to co-trimoxazole, the drug of choice when treating *S. maltophilia* infection. However, none of the isolates were categorized as intermediate or resistant based on the CLSI guidelines (2022). Increased resistance to all the tested antibiotics was exhibited by 28% of the isolates (Fig 1).

Mutation analysis of the PQ/CIP-resistant mutants

Five representative PQ/CIP-resistant isolates (KPQC3, KPQC13, KPQC22, KPQC24, and KPQC29) with different antibiotic susceptibility patterns (Fig 2A) were selected for the whole genome analysis. The MIC of CIP was 32 μg/ml for KPQC3 and KPQC13, 64 μg/ml for KPQC22, and 16 μg/ml KPQC24 and KPQC29. All the selected isolates showed increased resistance to the quinolones (nalidixic acid, norfloxacin, CIP, moxifloxacin, and levofloxacin) and azithromycin. KPQC3, KPQC13, KPQC22, and KPQC29 displayed increased resistance to the aminoglycosides (gentamicin, amikacin and netilmicin), and KPQC3, KPQC13, and KPQC29 showed increased resistance to meropenem (Fig 2A). KPQC3 and KPQC22 showed increased resistance to the cephalosporins (ceftazidime and cefoperazone). Increased resistance to co-trimoxazole was observed in KPQC3, KPQC22, and KPQC29.

The whole genome sequences of the mutants were aligned with those of the *S. maltophilia* K279a parental strains [2] available on the NCBI database (NC_010943.1). The mutations identified via the whole genome sequence analysis are summarized in Table 2. The frameshift mutations occurred in genes such as *smlt4073* (*smeT* encodes the transcription regulator in the TetR family), *smlt0009* (periplasmic protein TonB), *smlt0895* (putative 50S ribosomal subunit protein L1, *rplA*), and *smlt3608* (putative aconitase A, *acnA*). Stop-gained or nonsense mutations were detected in *smlt3910* (putative glycine betaine transporter 2, *opuD2*). An inframe insertion mutation was identified in *smlt0570* (putative hybrid histidine kinase/responsive regulator). Nonsynonymous SNPs were found in *smlt0082* (conserved hypothetical protein in the TIGR00266 family with an unknown function), *smlt0151* (putative glutamine synthetase), *smlt0276* (conserved hypothetical protein), *smlt2538* (conserved hypothetical protein containing a ferritin-like domain), *smlt3910* (putative glycine betaine transporter 2 *opuD2*), and *smlt4687* (23S rRNA (cytosine1962-C5)-methyltransferase). All the mutants had nonsynonymous SNPs in *smlt2678* (*mfsQ* encoding a major facilitator family efflux pump is important for resistance against quaternary ammonium compounds [22]). Furthermore, no mutations

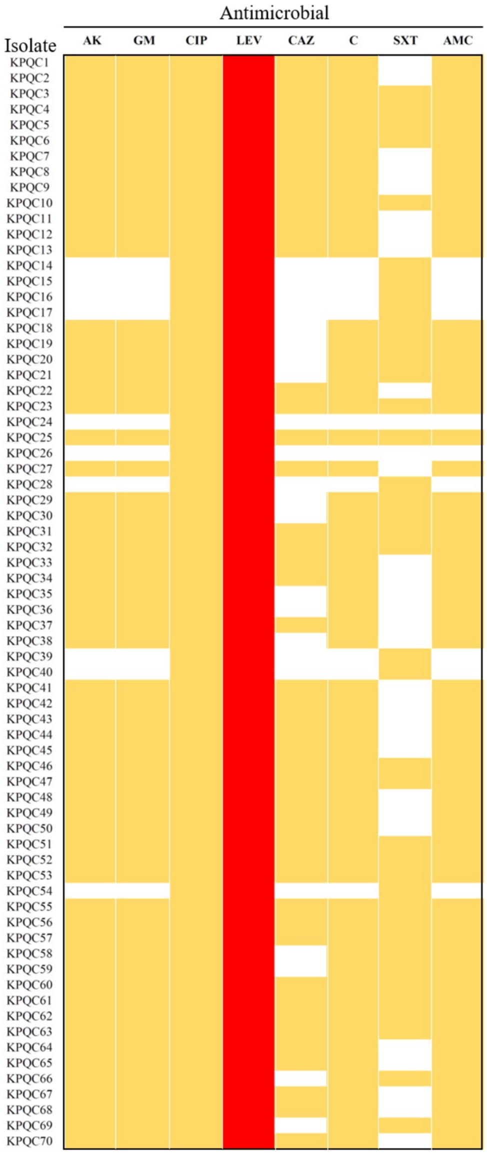

Fig 1. Antimicrobial susceptibility of the PQ/CIP-resistant isolates. The heat map shows the antimicrobial susceptibility patterns of 70 PQ/CIP-resistant isolates determined using the standard disk diffusion. Isolates that showed increased antibiotic resistance (smaller zone of inhibition ≥ 5 mm than the K279a wild-type) were marked in yellow, while colonies that are judged as resistant according to the CLSI guidelines (2022) were marked in red. AK, amikacin; GM, gentamicin; CIP, ciprofloxacin; LEV, levofloxacin; CAZ, ceftazidime; C, chloramphenicol; AMC, amoxicillin/clavulanic acid; SXT, co-trimoxazole.

https://doi.org/10.1371/journal.pone.0309525.g001

were observed in the genes that encoded the drug degrading/modifying enzymes, the multi-drug efflux pumps, or the transcriptional regulator SoxR.

Expression analysis of antimicrobial resistance genes in the PQ/CIP-resistant mutants

S. maltophilia carries multiple intrinsic antibiotic-resistant mechanisms, such as numerous chromosomally encoded efflux pumps, β-lactamases, and aminoglycoside-modifying enzymes.

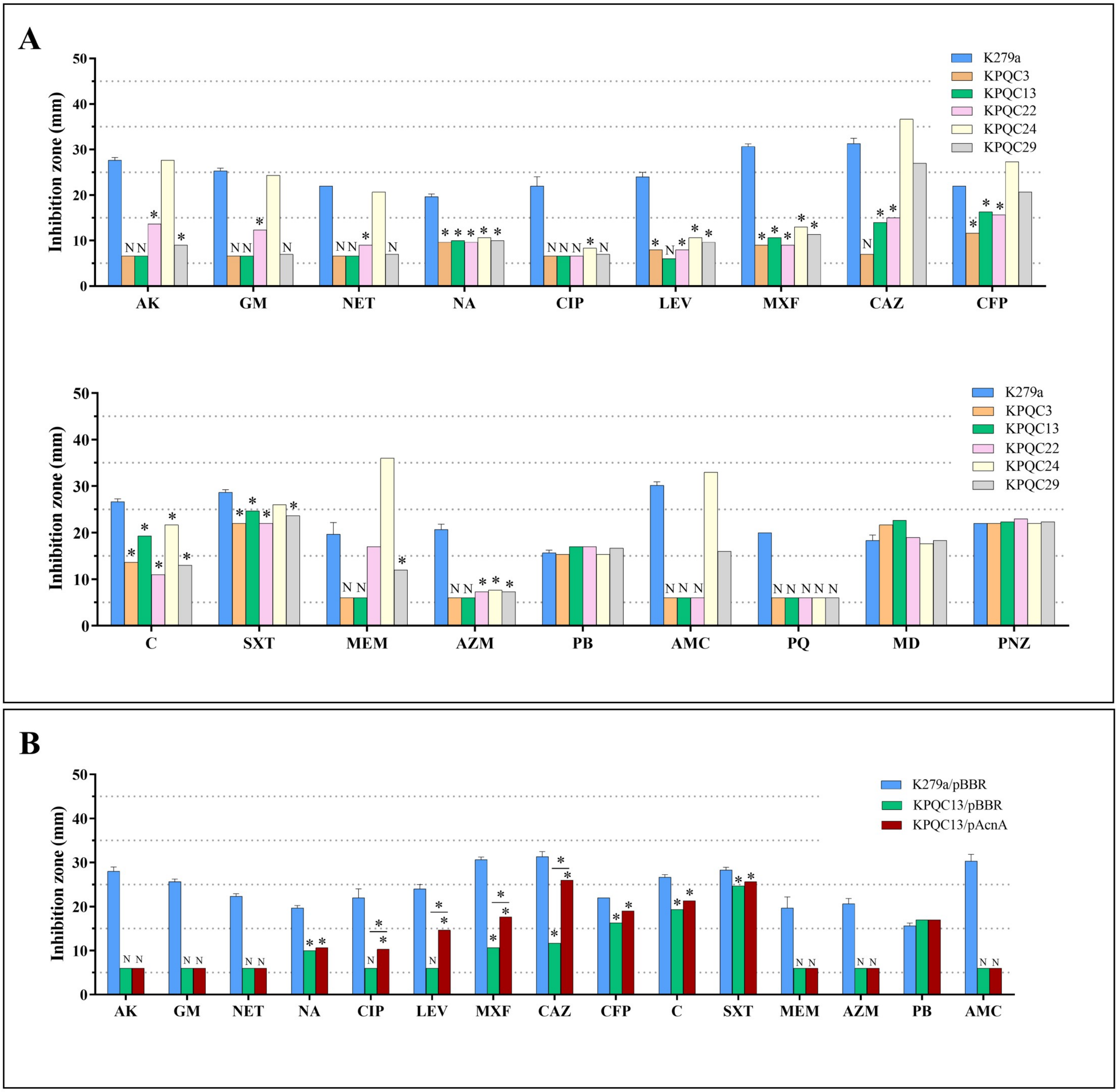

Fig 2. Antimicrobial susceptibility profiles of *S. maltophilia* strains. Antimicrobial susceptibility profiles of wild-type K279a, KPQC3, KPQC13, KPQC22, KPQC24, and KPQC29 (A); and K279a harboring pBBR1MSC-3 (K279a/pBBR) and KPQC13 harboring pAcnA (KPQC13/pAcnA) or pBBR1MCS-3 vector (KPQC13/pBBR) (B) were determined using the standard disk diffusion. AK, amikacin; GM, gentamicin; NET, netilmicin; NA. nalidixic acid; CIP, ciprofloxacin; LEV, levofloxacin; MXF, moxifloxacin; CAZ, ceftazidime; CFP, cefoperazone; C, chloramphenicol; SXT, co-trimoxazole; MEM, meropenem; AZM, azithromycin; PB, polymyxin B; AMC, amoxicillin/clavulanic acid. Paraquat (PQ), menadione (MD), and phenazine (PNZ) were the representatives of superoxide generators. N indicates no inhibition zone. Asterisk (*) in (A) indicates significant difference relative to K279a (p-value < 0.05, ANOVA and Dunnett's post-hoc test), while in (B) indicates significant difference between KPQC13/pBBR and KPQC13/pAcnA (p-value < 0.05, paired t-test).

https://doi.org/10.1371/journal.pone.0309525.g002

Table 2. Mutational analysis of the selected mutants.

Locus_tag	Type of mutation	Effect	Gene product	Strain
smlt0009	INS	frameshift_variant c.556_557dupCT p.Asp187fs	Periplasmic protein TonB (ExbB-ExbD) complex	KPQC3
smlt0082	SNV	nonsynonymous_variant c.989G>T p.Arg330Leu	conserved hypothetical protein	KPQC3
smlt0151	SNV	nonsynonymous_variant c.1186G>A p.Asp396Asn	GlnA,putative glutamine synthetase (glutamate—ammonia ligase)	KPQC24
smlt0276	SNV	nonsynonymous_variant c.191A>C p.Gln64Pro	conserved hypothetical protein	KPQC22
smlt0518	SNV	synonymous_variant c.768C>A p.Ile256Ile	putative transposase	KPQC29
smlt0570	INS	conservative_inframe_insertion c.1753_1761dupCCGGGCATG p.Pro585_Met587dup	putative sensor histidine kinase/response regulator fusion protein	KPQC3, KPQC22
smlt0895	INS	frameshift_variant c.687dupG p.Thr230fs	RplA (50S ribosomal subunit protein L1)	KPQC3
	SNV	nonsynonymous_variant c.125A>G p.Asp42Gly	RplA (50S ribosomal subunit protein L1)	KPQC13
smlt1466	SNV	synonymous_variant c.105G>T p.Val35Val	ATP binding protein	KPQC24
smlt1771	SNV	synonymous_variant c.186G>A p.Lys62Lys	Alkyl hydroperoxide reductase (AhpC)	KPQC22
smlt2538	SNV	nonsynonymous_variant c.61G>A p.Ala21Thr	hypothetical protein containing ferritin-like domain	KPQC3, KPQC13, KPQC22, KPQC29
smlt2678	SNV	nonsynonymous_variant c.953C>A p.Ala318Glu	MfsQ, major facilitator superfamily	KPQC3, KPQC29
	SNV	nonsynonymous_variant c.877C>G p.Gln293Glu	MfsQ, major facilitator superfamily	KPQC13, KPC22, KPC24
smlt3608	complex	frameshift_variant&nonsynonymous_variant c.800_808delGCGTGGTCGinsTCGGTCA p.Arg267fs	AcnA, (putative aconitase A or aconitate hydratase 1)	KPQC13
smlt3910	SNV	nonsynonymous_variant c.116T>A p.Met39Lys	OpuD2 (putative glycine betaine transporter 2)	KPQC13
	SNV	stop_gained c.1193G>A p.Trp398*	OpuD2 (putative glycine betaine transporter 2)	KPQC22
smlt3980	SNV	nonsynonymous_variant c.815C>T p.Ala272Val	putative LysR family transcription regulatory protein	KPQC24
smlt4073	DEL	frameshift_variant c.255delC p.Val86fs	SmeT, TetR family regulatory protein	KPQC24
smlt4687	SNV	nonsynonymous_variant c.481G>C p.Val161Leu	23S rRNA (cytosine1962-C5)-methyltransferase	KPQC24
**INTERGENIC REGION**				
position 2430153 position 2430178	SNV SNV	A>G T>C	intergenic region between *smlt2389* (putative phage protein) and *smlt2390* (transmembrane protein)	KPQC29
position 2436397	SNV	C>T	intergenic downstream of gene *smlt2399* (hypothetical protein) and *smlt2400* (hypothetical protein)	KPQC29

https://doi.org/10.1371/journal.pone.0309525.t002

We aimed to determine whether alterations in the antibiotic-resistant phenotypes of the PQ/CIP-resistant mutants were due to changes in the expressions of the genes that contribute to antibiotic resistance in *S. maltophilia*. The expression levels of the genes encoding efflux pumps (i.e., *smeABC*, *smeDEF*, *smeGH*, *smeIJK*, *smeOP*, *smeVWX*, *smeYZ*, *smaAB*, *smaCDEF*, *mfsA*, *smrA*, *macABCsm*, *smtcrA*, and *pmpM*), antibiotic-degrading or -modifying enzymes (*blaL1* (L1 β-lactamase), *blaL2* (L2 β-lactamase), *aac(6')-Iz* (aminoglycoside 6′-*N*-acetyltransferase), and *aph(3')-IIc* (aminoglycoside-3'- phosphotransferase)), and the quinolone resistance gene *smqnrB* [4,10,21,23–25] were determined using real-time RT-PCR. As shown in Table 3, relative to the K279a wild-type, KPQC3 showed high expression of *smeYZ* (34.4 ± 17.9 fold), *smaAB* (10.1 ± 4.1 fold), and *blaL2* (5.1 ± 2.4 fold). KPQC13 displayed increased expression of the genes encoding efflux pumps, that is, *smeYZ* (44.4 ± 12.2 fold), *smaAB* (6.1 ± 2.5 fold), *smaC-DEF* (4.4 ± 2.5 fold), and *smtcrA* (4.9 ± 1.5 fold), while KPQC22 expressed elevated levels of *smaCDEF* (6.0 ± 4.0 fold), *blaL2* (5.6 ± 1.9 fold), and *aph(3')-IIc* (3.2 ± 0.7 fold). KPQC24

Table 3. Expression profiles of the antimicrobial resistance genes in the *S. maltophilia* mutants.

Genes	Locus tag	Product	Antibiotic resistance	Relative expression (fold)				
				KPQC3	KPQC13	KPQC22	KPQC24	KPQC29
smeABC	Smlt4474-6	RND-type efflux pump	Aminoglycosides, β-lactams and quinolones	0.8 ± 0.3	1.1 ± 0.4	0.8 ± 0.1	1.5 ± 0.3	1.3 ± 0.7
smeDEF	Smlt4070-2	RND-type efflux pump	C, TE, SXT and quinolones	1.5 ± 0.6	1.5 ± 0.3	1.5 ± 0.3	5.7 ± 3.9*	1.8 ± 0.6
smeGH	Smlt3170-1	RND-type efflux pump	Quinolones, macrolides, C, TE, ceftazidime	0.7 ± 0.2	1.3 ± 0.5	0.7 ± 0.1	1.0 ± 0.3	1.0 ± 0.2
smeIJK	Smlt4279-81	RND-type efflux pump	TE, CIP and aminoglycosides	0.9 ± 0.1	1.8 ± 0.9	1.0 ± 0.1	1.6 ± 0.4	0.9 ± 0.1
smeOP	Smlt3925-4	RND-type efflux pump	Aminoglycosides, macrolides and SXT	1.0 ± 0.2	1.2 ± 0.2	0.9 ± 0.2	1.9 ± 0.8	1.1 ± 0.1
smeVWX	Smlt1830-3	RND-type efflux pump	C and quinolones	0.6 ± 0.1	0.7 ± 0.1	0.3 ± 0.1	0.9 ± 0.2	5.7 ± 0.6*
smeYZ	Smlt2201-2	RND-type efflux pump	Aminoglycosides and SXT	34.4 ± 17.9*	44.4 ± 12.2*	1.5 ± 0.5	2.1 ± 0.7	30.5 ± 8.4*
smrA	Smlt1471	ABC-type efflux pump	Fluoroquinolones and tetracycline	1.2 ± 0.1	1.1 ± 0.1	1.4 ± 0.3	1.1 ± 0.1	1.1 ± 0.1
macABCsm	Smlt2642-3	ABC-type efflux pump	Aminoglycosides, macrolides and polymyxins	1.4 ± 0.7	1.0 ± 0.1	1.0 ± 0.3	1.7 ± 1.0	1.2 ± 0.3
smaAB	Smlt2642-3	ABC-type efflux pump	Aminoglycosides	10.1 ± 4.1*	6.1 ± 2.5*	2.3 ± 1.8	1.0 ± 0.2	2.0 ± 1.3
smaCDEF	Smlt1651-4	ABC-type efflux pump	Fluoroquinolones	2.1 ± 0.8	4.4 ± 2.5*	6.0 ± 4.0*	5.4 ± 1.1*	6.1 ± 3.3*
mfsA	Smlt1083	MFS-type efflux pump	Fluoroquinolones	1.1 ± 0.6	1.8 ± 0.5	0.6 ± 0.1	1.4 ± 1.0	0.8 ± 0.1
smtcrA	Smlt1069	MFS-type efflux pump	Quinolones	1.2 ± 0.5	4.9 ± 1.5*	0.8 ± 0.1	0.9 ± 0.1	1.0 ± 0.1
pmpM	Smlt1381	MATE-type efflux pump	Fluoroquinolones	1.1 ± 0.4	2.4 ± 0.9*	1.7 ± 0.8	0.7 ± 0.2	0.8 ± 0.4
blaL1	Smlt2667	Class B metallo L1 β-lactamase	β-lactams	2.1 ± 0.9	1.1 ± 0.4	2.5 ± 1.4	1.4 ± 0.4	0.9 ± 0.3
blaL2	Smlt3722	Class A L2 β-lactamase	β-lactams	5.1 ± 2.4*	1.4 ± 0.4	5.6 ± 1.9*	0.9 ± 0.1	0.9 ± 0.1
aph (3') *IIc*	Smlt2120	aminoglycoside-3'-phosphotransferase	Aminoglycosides	1.5 ± 0.3	1.7 ± 0.2	3.2 ± 0.7*	2.2 ± 0.9*	1.2 ± 0.3
aac (6')-Iz	Smlt3615	Aminoglycoside 6′-*N*-acetyltransferase	Aminoglycosides	0.8 ± 0.3	1.0 ± 0.4	0.9 ± 0.2	1.1 ± 0.1	1.1 ± 0.2
smqnrB	Smlt1071	Quinolone resistance protein	quinolones	1.3 ± 0.3	1.1 ± 0.2	1.4 ± 0.6	1.3 ± 0.5	0.9 ± 0.1
sodA1	Smlt2828	Mn-SOD	ND	0.6 ± 0.1	1.9 ± 0.4	1.4 ± 1.2	1.0 ± 0.0	0.9 ± 0.6
sodA2	Smlt3238	Mn-SOD	ND	0.3 ± 0.1	0.6 ± 0.5	0.8 ± 0.3	1.4 ± 0.8	0.9 ± 0.1
sodB	Smlt1616	Fe-SOD	ND	1.3 ± 0.5	1.6 ± 0.7	1.1 ± 0.1	1.1 ± 0.2	1.0 ± 0.1
sodC1	Smlt0160	Cu, Zn-SOD	ND	1.1 ± 0.2	1.1 ± 0.5	0.9 ± 0.3	0.9 ± 0.2	0.9 ± 0.1
sodC2	Smlt0161	Cu, Zn-SOD	ND	1.1 ± 0.8	1.0 ± 0.3	0.9 ± 0.1	1.1 ± 0.3	0.8 ± 0.1

Data represent the mean ± SD of fold change relative to the expression of K279a from three replicated experiments. C, chloramphenicol; TE, tetracycline; SXT, co-trimoxazole; ND, no data. Asterisk (*) indicates significant difference compared to K279a according to one-way ANOVA, followed by Dunnett's post-hoc test ($p < 0.05$).

https://doi.org/10.1371/journal.pone.0309525.t003

showed enhanced expression of *smeDEF* (5.7 ± 3.9 fold) and *smaCDEF* (5.4 ± 1.1 fold), and KPQC29 expressed high levels of *smeYZ* (30.5 ± 8.4 fold), *smeVWX* (5.7 ± 0.6 fold), and *smaCDEF* (6.1 ± 3.3 fold). The antibiotic phenotypes of the PQ/CIP-resistant mutants therefore arose in part from the enhanced expression of the antibiotic resistance genes.

The *S. maltophilia* K279a genome [2] contains five coding sequences for SOD, namely, Smlt0160 (*sodC1* encoding Cu-ZnSOD), Smlt0161 (*sodC2* encoding Cu-ZnSOD), Smlt1616 (*sodB* encoding FeSOD), Smlt2828 (*sodA1* encoding MnSOD), and Smlt3238 (*sodA2* encoding MnSOD). We also determined the expression levels of these *sod* genes. No significant changes in the expression levels of any of the *sod* genes relative to the K279a wild-type were detected in any of the tested PQ/CIP-resistant mutants (Table 2). The PQ-resistant phenotype of the mutants is thus unlikely due to the increased expression of superoxide dismutases. Moreover, the susceptibility levels of the selected PQ/CIP-resistant mutants against the redox cycling agents menadione, phenazine, and PQ were determined using disk diffusion assays. None of

the tested mutants (KPQC3, KPQC13, KPQC22, KPQC24, and KPQC29) showed any alterations in their susceptibility levels against menadione and phenazine (Fig 2A).

Complementation of KPQC13 with wild-type *acnA* (*smlt3608*)

The mutant KPQC13 showed a frameshift mutation of *smlt3608* (*acnA*) encoding aconitase A, which shares a 42% amino acid identity with *Escherichia coli* AcnA [26]. To validate the role of *S. maltophilia acnA* in antimicrobial susceptibility, a full-length *acnA* gene from K279a was PCR amplified and cloned into a pBBR1MCS-3 [20] expression plasmid vector, which generated pAcnA. The antibiotic susceptibility profile of KPQC13 harboring pAcnA (KPQC13/pAcnA) was determined and compared with that of the K279a wild-type and KPQC13 harboring empty vector. The expression of AcnA partially complemented the antimicrobial resistance phenotype of KPQC13 against CIP, levofloxacin, moxifloxacin, and ceftazidime (Fig 2B).

Discussion

In this study, the sequential exposure of *S. maltophilia* K279a to PQ resulted in the formation of mutants that showed increased resistance to PQ. Around 17.5% of the PQ-resistant isolates could resist CIP (at a concentration of 16 μg/ml) and levofloxacin. As a redox cycling compound, PQ undergoes enzymatic one-electron reduction to generate a transient PQ radical, which can be reoxidized by molecular oxygen (O_2) to continuously produce superoxide anion, a primary oxygen radical [9]. Superoxide anion is a highly reactive species that is able to trigger a cascade of ROS, such as H_2O_2, and hydroxyl radicals, which cause oxidative damage to biomolecules. The nucleotide pool is also a target of ROS, and the incorporation of oxidized nucleotides into DNA leads to an increase in mutation frequency [27]. The exposure of *S. maltophilia* to PQ would therefore induce mutations, which lead to resistance phenotypes to PQ as well as antibiotics.

SoxR mediated the interplay between the oxidative stress response and antimicrobial resistance in *S. maltophilia*. SoxR regulates the genes that alleviate PQ toxicity, including *mfsA* (MFS efflux transporter), *sodA1* (SOD), and those that are involved as multidrug transporters (*mfsA* and *smeVWX*) [9,11,28]. Mutations of *soxR* have been reported to be associated with the upregulation of genes in regulons [29]. Unexpectedly, neither the mutations of *soxR* nor the promoter region of *soxR* were detected in any of the selected PQ/CIP-resistant mutants. Our expression analysis revealed that the levels of *mfsA* and *sodA1* transcription were not altered in any of the PQ/CIP-resistant mutants relative to the K279a wild-type. However, the KPQC29 mutant showed increased levels of *smeVWX* expression (4.3 ± 2.0 fold). Upregulation of *smeVWX* is at least in part responsible for increased resistance to quinolones and chloramphenicol in the KPQC29 mutant. Three regulators (i.e., SmeRv, AzoR, and SoxR) are involved in the regulation of *smeVWX* expression. SmeRv and AzoR directly control the expression of *smeVWX* as positive and negative regulators, respectively, while oxidized SoxR upregulates *smeRv* expression [28,30,31]. We found that increased *smeVWX* expression in the KPQC29 mutant was not due to mutations of the *smeRv*, *azoR*, and *soxR* genes or their promoter regions. Nevertheless, changes in the levels of the SmeRv and AzoR proteins in the KPQC29 mutant could not be ruled out.

The expression of the other *sod* genes (i.e., *sodA2*, *sodB*, *sodC1* and *sodC2*) were also unchanged (Table 2). The PQ-resistant phenotype of the mutant thus did not rise due to increased levels of the SODs and MfsA efflux pump. This is supported by the finding that none of the tested PQ/CIP resistance showed any increased resistance against the other redox cycling agents/superoxide generators, including menadione and phenazine (Fig 2B). Mechanisms in *S. maltophilia* other than SODs and MfsA are therefore responsible for PQ resistance.

Mutations that cause changes in the genes involved in the regulation and expression of functional genes contributing to cell permeability, antibiotic resistance mechanisms, efflux transporters, RNA polymerase subunits, and cell metabolism generally explain the resistance of bacteria to antibiotics [32–34]. Whole genome sequence analysis of the selected PQ/CIP-resistant mutants revealed mutations with potential effects on antibiotic resistance, such as the genes encoding SmeT (*smlt4073*) the transcriptional regulator in the TetR family, the periplasmic protein TonB (*smlt0009*), and the 50S ribosomal subunit protein L1 (*smlt0895*, *rplA*). SmeT negatively regulates the expression of *smeDEF*, which encodes the multidrug efflux pumps belonging to the resistance-nodulation-cell-division (RND) family [35]. Mutations of SmeT lead to constitutively high expression of *smeDEF*, which is associated with increased resistance to co-trimoxazole, macrolide erythromycin, chloramphenicol, tetracyclines, and quinolones [36,37]. In our study, the expression analysis revealed that the KPQC24 mutant, in which *smeT* was mutated, expressed elevated levels of *smeDEF* (5.7 ± 3.9 fold) relative to its parental wild-type K279a. However, KPQC24 was highly resistant to quinolones (nalidixic acid, CIP, levofloxacin and moxifloxacin) and macrolide (azithromycin) but not to co-trimoxazole, and chloramphenicol.

KPQC3 contained a frameshift mutation of *smlt0009*, which encodes the periplasmic membrane protein TonB. TonB is an energy transducing protein of the Ton complex, which consists of TonB, ExbB, and ExbD. The Ton complex uses the proton motive force at the inner membrane to couple energy to the outer membrane transporters to facilitate the uptake of rare nutrients, including iron and cobalamine, into the periplasm [38]. Disruption of TonB has been shown to render *S. maltophilia* resistant to the siderophore-conjugated antibiotics that require Ton complex-mediated transport, such as β–lactams and fluoroquinolones [39]. The increased resistance of KPQC3 to cephalosporins, carbapenems, and fluroquinolone is thus in part due to the mutation of TonB, which results in a lower uptake of antibiotics.

We observed mutations of *rplA* in KPQC3 and KPQC13 mutants. Mutations of the 50S ribosomal protein L1 (RplA) have been observed in the gentamicin resistance mutants of *S. maltophilia* K279a, which were selected in a laboratory; mutations of *rplA* led to the activation of aminoglycoside efflux pump SmeYZ production, which thereby increased resistance to aminoglycosides [40]. Similarly, in this study, both mutants expressed high levels of *smeYZ* (34.4 ± 17.9 for KPQC3 and 44.4 ± 12.2 for KPQC13). Mutations of *rplA* leading to high expression of SmeYZ would contribute to increased resistance to aminoglycoside antibiotics with KPQC3 and KPQC13 mutants. It has been proposed that aminoglycosides, the drugs that target the A-site of 16S ribosomal RNA of the 30S ribosome, are potential drivers of the selection of strains with overproduced SmeYZ that occurs due to *rplA* mutations in *S. maltophilia* [40]. A more recent study illustrated that treating K279a with a sublethal level of CIP can also induce *rplA* mutation [19]. Accordingly, mutations of *rplA* can be induced not only through exposure to aminoglycosides and CIP but also to PQ. This acquired resistance may be one of the important factors rendering *S. maltophilia* resistant to aminoglycosides because mutations of RplA associated with the overproduction of SmeYZ exist in various clinical isolates [40]. It is of note that the contribution of SmeYZ to the virulence of *S. maltophilia* in a mouse model has been reported [41]. Moreover, KPQC29 was also expressed high level of *smeYZ* (30.5 ± 8.4 fold) without mutations of ribosomal proteins or its transcriptional regulators, two-component regulatory system SmeSyRy (Smlt2200 and Smlt2199) [42] and SmaRS (Smlt2645 and Smlt2646) [21]. The reason for upregulation of SmeYZ in KPQC29 is unknown. However, KPQC29 carried two mutations on *mfsQ* (*smlt2678*) and *smlt2538* encoding hypothetical protein containing ferritin-like domain. MfsQ has been shown to play no roles on antibiotic resistance of *S. maltophilia* K279a and is regulated by MfsR, a TetR type transcriptional regulator [22,43]. Despite containing ferritin-like domain, *smlt2538* was not a member of genes

contributing to iron homeostasis systems identified in *S. maltophilia* K279a [44]. The physiological function of *smlt2538* in both iron homeostasis and antibiotic resistance is under investigation. Notably, the high expression of the SmeYZ aminoglycoside efflux pump in KPQC3, KPQC13, and KPQC29 mutants relative to the K279a wild-type (34.4 ± 17.9, 44.4 ± 12.2, and 30.5 ± 8.4 fold, respectively) was associated with enhanced resistance to amikacin, gentamicin, and netilmicin. This is in contrast to the lower expression levels observed in KPQC22 and KPQC24 (1.5 ± 0.5 and 2.1 ± 0.7 fold, respectively), as shown in Table 3 and Fig 2.

The KPQC13 mutant showed frameshift mutation of *acnA* (*smlt3608*) encoding aconitase A. Aconitase, a [4Fe-4S] cluster containing enzyme, catalyzes the reversible and stereo-specific isomerization of citrate and isocitrate via *cis*-aconitate in the tricarboxylic acid (TCA) cycle. *E. coli* produces two aconitases, AcnA. a stationary-phase enzyme, that can be induced by iron and oxidative stress, and AcnB, a major enzyme synthesized during exponential growth [45]. Moreover, the AcnA and AcnB apo-proteins function as post-transcription regulator by interacting with the target mRNAs [46]. The finding that complementation of KPQC13 with pAcnA plasmid could partially diminish the increased antibiotic resistance of the KPQC13 mutant against fluoroquinolone antibiotics (CIP, levofloxacin, and moxifloxacin) and cephalosporin (ceftazidime) indicates the involvement of AcnA in the antibiotic resistance of *S. maltophilia*. The linkage between aconitase and antibiotic resistance has recently been reported in *Vibrio cholerae*, where a lack of AcnB increased its tolerance to the antibiotics that target protein synthesis, including tetracycline, erythromycin, and chloramphenicol [47]. It has been reported that ROS plays a role in the antibiotic-mediated killing of bacteria, in particular those belonging to aminoglycosides, quinolones, and β-lactam [48]. The TCA cycle increases ROS production by generating the reducing equivalents NADH and $FADH_2$, which deliver their electrons to the electron transport chain; this catalytic activity is believed to be triggered by treatment with antibiotics [48]. Thus, lower TCA cycle activity, for example, disruption of aconitase genes would reduce the level of post-antibiotic ROS production, thus rendering it less susceptible to the antibiotic [47]. The mutation of the aconitase gene *acnA* therefore contributes partly to the increased resistance to quinolones observed in the KPQC13 mutant. Moreover, the ectopic expression of *acnA* from pAcnA was unable to fully complement the increased antibiotic resistance phenotypes (fluoroquinolones and ceftazidime) of KPQC13 to wild-type levels. This might be due to the presence of mutations in other genes in KPQC13, such as *rplA* and *opuD2*, which may also influence antimicrobial resistance. Alternatively, the use of pBBR1MCS-3, a medium-copy-number plasmid vector, in a complementation experiment could be a contributing factor. High expression of genes on pAcnA, including *acnA* and tetracycline resistance genes, may adversely affect KPQC13's physiology. Utilizing a single-copy complementing system might yield more definitive results.

Mutations of *opuD2* (*smlt3910*) encoding the putative glycine betaine transporter were detected in KPQC13 and KPQC22. The glycine betaine transporter belongs to the betaine-choline-carnitine transporter (BCCT) family. It is involved in the uptake of a compatible solute glycine betaine, which is a small, uncharged organic compound that functions as an osmoprotectant to maintain cell permeability and volume during high salinity stress [49]. To our knowledge, the role of the glycine betaine transporter in antibiotic resistance is as yet unknown and is being investigated.

KPQC24 had a point mutation of *glnA* (*smlt0151*) encoding putative glutamine synthetase type I, an enzyme that catalyzes the reaction that produces glutamine from the condensation of L-glutamate and ammonia. Glutamine is used as a nitrogen donor for synthesizing nitrogen-containing molecules, such as purine and pyrimidine [50]. *S. maltophilia* K279a possesses two coding sequences, *smlt0151* and *smlt1580*, encoding putative glutamine synthetases [2]. Metabolite analysis has revealed that glutamine is repressed in multidrug-resistant

uropathogenic *E. coli* [51]. Exogenous glutamine potentiates the killing activity of β-lactam, aminoglycoside, quinolone, and tetracycline against *E. coli* and promotes the influx of ampicillin, which leads to the accumulation of the antibiotic [51]. Glutamine-potentiated ampicillin-mediated bacterial killing has been observed in other gram negative bacteria, including *Pseudomonas aeruginosa*, *Acinetobacter baumannii*, *Klebsiella pneumonia*, and *Vibrio parahaemolyticus* [51]. In KPQC24, the point mutation of GlnA occurred at D396N, which is located on the adenylation loop and regulates glutamine synthetase activity via the adenylation of a tyrosine residue. The conserved adenylation loop NLYDLP [52] corresponded to the D_{396}LYDLP of *S. maltophilia* GlnA. The D396N mutation would therefore make the mutant's adenylation loop match the conserved NLYDLP domain perfectly, and it is thus unlikely to affect the enzymatic activity of GlnA.

Altogether, our results indicate that increased antimicrobial resistance seems to be in harmony with the upregulation of the genes responsible for the antibiotic resistance of the *S. maltophilia* PQ/CIP-resistant mutants. The development of multidrug-resistant mutants due to PQ exposure highlights the risk that improper use of the herbicide PQ can contribute to antibiotic resistance problems.

Furthermore, there are some limitations in this study. Selecting a larger number of PQ/CIP-resistant mutants may reveal more novel genes contributing to antibiotic resistance in *S. maltophilia*. We have identified mutations in genes that have never been reported for their involvement in antibiotic resistance in *S. maltophilia*, such as *opuD2*, *acnA*, and *glnA*. Further investigations are therefore required to elucidate their functions.

Conclusion

In this study, we showed that exposure of *S. maltophilia* K279a to PQ can induce the development of mutant strains with increased antimicrobial resistance to CIP, clinically important quinolone levofloxacin, and other quinolones. Simultaneously increased resistance of the PQ/CIP-resistant mutants to aminoglycosides, cephalosporins, carbapenems, chloramphenicol, azithromycin, and co-trimoxazole was observed. Mutations of the SmeT regulator and ribosomal protein L1 led to the increased production of antibiotic resistance proteins, mainly drug efflux pumps. The mutation of the AcnA enzyme in the central metabolism rendered it resistant to antibiotics, including fluoroquinolones and ceftazidime. The extensive use and poor management of the herbicide PQ may stimulate the development of antimicrobial-resistant strains of *S. maltophilia* in soil and aquatic environments. It is recognized that environments, particularly aquatic ones, act as reservoirs and transmission routes for the dissemination of pathogens to humans [53]. This raises concerns that the misuse of PQ could increase the risk of developing antibiotic resistance, especially in *S. maltophilia*.

Supporting information

S1 Fig. The melting curves of the amplified antibiotic resistance genes. Real-time RT-PCR was used to assess the expression levels of potential antibiotic resistance genes in both wild-type and mutant strains of *S. maltophilia* K279a. The melting curves from a representative real-time RT-PCR experiment for all amplified genes are shown.
(PPTX)

S2 Fig. Confirmation of the sequence of the *acnA* gene inserted in pAcnA by DNA sequencing. Pairwise alignment between the *acnA* gene (*smlt3608*) and the insert in pAcnA was conducted using the ClustalW algorithm (https://www.genome.jp/tools-bin/clustalw). An

asterisk (*) indicates identical sequences. The start (ATG) and stop (TGA) codons are in bold font.
(PPTX)

S1 Table. The raw data of the expression levels of antibiotic resistance genes in *S. maltophilia* K279a and its mutant stains (Table 3). The expression levels (fold) of antibiotic resistance genes in *S. maltophilia* mutant strains relative to the K279a wild-type from three independent real-time RT-PCR experiments are shown.
(XLSX)

S2 Table. The raw data of the disk diffusion assays (Fig 2). The inhibition zone (mm) against various antibiotics and oxidants for *S. maltophilia* strains from three independent experiments are shown.
(XLSX)

Author Contributions

Conceptualization: Nisanart Charoenlap, Paiboon Vattanaviboon.

Data curation: Veerakit Vanitshavit, Nisanart Charoenlap.

Formal analysis: Veerakit Vanitshavit, Nisanart Charoenlap, Punyawee Dulyayangkul.

Funding acquisition: Nisanart Charoenlap, Skorn Mongkolsuk, Paiboon Vattanaviboon.

Investigation: Veerakit Vanitshavit, Ratiboot Sallabhan, Wirongrong Whangsuk, Kisana Bhinija, Punyawee Dulyayangkul.

Methodology: Veerakit Vanitshavit, Nisanart Charoenlap, Paiboon Vattanaviboon.

Resources: Skorn Mongkolsuk.

Software: Veerakit Vanitshavit, Punyawee Dulyayangkul.

Supervision: Nisanart Charoenlap, Skorn Mongkolsuk, Paiboon Vattanaviboon.

Validation: Veerakit Vanitshavit, Paiboon Vattanaviboon.

Visualization: Nisanart Charoenlap, Punyawee Dulyayangkul.

Writing – original draft: Paiboon Vattanaviboon.

Writing – review & editing: Nisanart Charoenlap, Paiboon Vattanaviboon.

References

1. Brooke JS. Advances in the Microbiology of Stenotrophomonas maltophilia. Clin Microbiol Rev. 2021; 34(3):e0003019. https://doi.org/10.1128/CMR.00030-19 PMID: 34043457.
2. Crossman LC, Gould VC, Dow JM, Vernikos GS, Okazaki A, Sebaihia M, et al. The complete genome, comparative and functional analysis of Stenotrophomonas maltophilia reveals an organism heavily shielded by drug resistance determinants. Genome Biol. 2008; 9(4):R74. https://doi.org/10.1186/gb-2008-9-4-r74 PMID: 18419807.
3. Gil-Gil T, Martinez JL, Blanco P. Mechanisms of antimicrobial resistance in Stenotrophomonas maltophilia: a review of current knowledge. Expert Rev Anti Infect Ther. 2020; 18(4):335–47. https://doi.org/10.1080/14787210.2020.1730178 PMID: 32052662.
4. Sanchez MB. Antibiotic resistance in the opportunistic pathogen Stenotrophomonas maltophilia. Front Microbiol. 2015; 6:658. https://doi.org/10.3389/fmicb.2015.00658 PMID: 26175724.
5. Stuart AM, Merfield CN, Horgan FG, Willis S, Watts MA, Ramirez-Munoz F, et al. Agriculture without paraquat is feasible without loss of productivity-lessons learned from phasing out a highly hazardous herbicide. Environ Sci Pollut Res Int. 2023. https://doi.org/http://doi.org/10.1007/s11356-022-24951-0 PMID: 36622585.

6. Farrington JA, Ebert M, Land EJ, Fletcher K. Bipyridylium quaternary salts and related compounds. V. Pulse radiolysis studies of the reaction of paraquat radical with oxygen. Implications for the mode of action of bipyridyl herbicides. Biochim Biophys Acta. 1973; 314(3):372–81. https://doi.org/10.1016/0005-2728(73)90121-7 PMID: 4751237.
7. Nazish T, Huang YJ, Zhang J, Xia JQ, Alfatih A, Luo C, et al. Understanding paraquat resistance mechanisms in Arabidopsis thaliana to facilitate the development of paraquat-resistant crops. Plant Commun. 2022; 3(3):100321. https://doi.org/10.1016/j.xplc.2022.100321 PMID: 35576161.
8. EPA U. Memorandum. Paraquat: Preliminary ecological risk assessment for registration review. United States Environmental Protection Agency, Washington, D.C. 2019.
9. Srijaruskul K, Charoenlap N, Namchaiw P, Chattrakarn S, Giengkam S, Mongkolsuk S, et al. Regulation by SoxR of mfsA, which encodes a major facilitator protein involved in paraquat resistance in Stenotrophomonas maltophilia. PLoS One. 2015; 10(4):e0123699. https://doi.org/10.1371/journal.pone.0123699 PMID: 25915643.
10. Vattanaviboon P, Dulyayangkul P, Mongkolsuk S, Charoenlap N. Overexpression of Stenotrophomonas maltophilia major facilitator superfamily protein MfsA increases resistance to fluoroquinolone antibiotics. J Antimicrob Chemother. 2018; 73(5):1263–6. https://doi.org/10.1093/jac/dky024 PMID: 29462315.
11. Jair HW, Lu HF, Huang YW, Pan SY, Lin IL, Huang HH, et al. Roles of the two-MnSOD System of Stenotrophomonas maltophilia in the alleviation of superoxide stress. Int J Mol Sci. 2019; 20(7). https://doi.org/10.3390/ijms20071770 PMID: 30974814.
12. Wang G, Wilson TJ, Jiang Q, Taylor DE. Spontaneous mutations that confer antibiotic resistance in Helicobacter pylori. Antimicrob Agents Chemother. 2001; 45(3):727–33. https://doi.org/10.1128/AAC.45.3.727-733.2001 PMID: 11181351.
13. Dulyayangkul P, Charoenlap N, Srijaruskul K, Mongkolsuk S, Vattanaviboon P. Major facilitator superfamily MfsA contributes to multidrug resistance in emerging nosocomial pathogen Stenotrophomonas maltophilia. J Antimicrob Chemother. 2016; 71(10):2990–1. https://doi.org/10.1093/jac/dkw233 PMID: 27334664.
14. Wood GL, Washington JA. Antibacterial Susceptibility Tests: Dilution and Disk Diffusion Methods. In Murray P.R (ed.): Manual of Clinical Microbiology. Washington DC: ASM Press;1327–1341. 1995.
15. CLSI. Performance standards for antimicrobial susceptibility testing, M100, 32nd ed. Clinical and Laboratory Standards Institute, Wayne, PA. 2022.
16. Bolger AM, Lohse M, Usadel B. Trimmomatic: a flexible trimmer for Illumina sequence data. Bioinformatics. 2014; 30(15):2114–20. https://doi.org/10.1093/bioinformatics/btu170 PMID: 24695404.
17. Andrews S. FastQC: a quality control tool for high throughput sequence data. Available online at: http://www.bioinformatics.babraham.ac.uk/projects/fastqc. 2012.
18. Seemann T. Snippy: fast bacterial variant calling from NGS reads. Available online at https://github.com/tseemann/snippy. 2015.
19. Chatree Y, Charoenlap N, Vanitshavit V, Ruangrassamee P, Mongkolsuk S, Vattanaviboon P. Induction of antimicrobial resistance of Stenotrophomonas maltophilia by exposure to nonlethal levels of antibiotics. Microb Drug Resist. 2023; 29(4):115–26. https://doi.org/10.1089/mdr.2022.0202 PMID: 36897754.
20. Kovach ME, Elzer PH, Hill DS, Robertson GT, Farris MA, Roop RM, et al. Four new derivatives of the broad-host-range cloning vector pBBR1MCS, carrying different antibiotic-resistance cassettes. Gene. 1995; 166(1):175–6. https://doi.org/10.1016/0378-1119(95)00584-1 PMID: 8529885.
21. Dulyayangkul P, Calvopina K, Heesom KJ, Avison MB. Novel mechanisms of efflux-mediated levofloxacin resistance and reduced amikacin susceptibility in Stenotrophomonas maltophilia. Antimicrob Agents Chemother. 2020; 65(1):e01284–20. https://doi.org/10.1128/AAC.01284-20 PMID: 33139281.
22. Chittrakanwong J, Charoenlap N, Mongkolsuk S, Vattanaviboon P. mfsQ encoding an MFS efflux pump mediates adaptive protection of Stenotrophomonas maltophilia against benzalkonium chloride. Can J Microbiol. 2021; 67(6):491–5. https://doi.org/10.1139/cjm-2020-0341 PMID: 33151759.
23. McCutcheon JG, Dennis JJ. The potential of phage therapy against the emerging opportunistic pathogen Stenotrophomonas maltophilia. Viruses. 2021; 13(6). https://doi.org/10.3390/v13061057 PMID: 34204897.
24. Chang YC, Tsai MJ, Huang YW, Chung TC, Yang TC. SmQnrR, a DeoR-type transcriptional regulator, negatively regulates the expression of Smqnr and SmtcrA in Stenotrophomonas maltophilia. J Antimicrob Chemother. 2011; 66(5):1024–8. https://doi.org/10.1093/jac/dkr049 PMID: 21393212.
25. Youenou B, Favre-Bonte S, Bodilis J, Brothier E, Dubost A, Muller D, et al. Comparative genomics of environmental and clinical Stenotrophomonas maltophilia strains with different antibiotic resistance profiles. Genome Biol Evol. 2015; 7(9):2484–505. https://doi.org/10.1093/gbe/evv161 PMID: 26276674.

26. Prodromou C, Haynes MJ, Guest JR. The aconitase of Escherichia coli: purification of the enzyme and molecular cloning and map location of the gene (acn). J Gen Microbiol. 1991; 137(11):2505–15. https://doi.org/10.1099/00221287-137-11-2505 PMID: 1838390.

27. AbdulSalam SF, Thowfeik FS, Merino EJ. Excessive reactive oxygen species and exotic DNA lesions as an exploitable liability. Biochemistry. 2016; 55(38):5341–52. https://doi.org/10.1021/acs.biochem.6b00703 PMID: 27582430.

28. Wu CJ, Chiu TT, Lin YT, Huang YW, Li LH, Yang TC. Role of smeU1VWU2X operon in alleviation of oxidative stresses and occurrence of sulfamethoxazole-trimethoprim-resistant mutants in Stenotrophomonas maltophilia. Antimicrob Agents Chemother. 2018; 62(2). https://doi.org/10.1128/AAC.02114-17 PMID: 29203487.

29. Nunoshiba T, Demple B. A cluster of constitutive mutations affecting the C-terminus of the redox-sensitive SoxR transcriptional activator. Nucleic Acids Res. 1994; 22(15):2958–62. https://doi.org/10.1093/nar/22.15.2958 PMID: 8065907.

30. Chen CH, Huang CC, Chung TC, Hu RM, Huang YW, Yang TC. Contribution of resistance-nodulation-division efflux pump operon smeU1-V-W-U2-X to multidrug resistance of Stenotrophomonas maltophilia. Antimicrob Agents Chemother. 2011; 55(12):5826–33. https://doi.org/10.1128/AAC.00317-11 PMID: 21930878.

31. Lu HF, Tsai YC, Li LH, Lin YT, Yang TC. Role of AzoR, a LysR-type transcriptional regulator, in SmeVWX pump-mediated antibiotic resistance in Stenotrophomonas maltophilia. J Antimicrob Chemother. 2021; 76(9):2285–93. https://doi.org/10.1093/jac/dkab203 PMID: 34151959.

32. Ferreira MA, Pereira ML, Dos Santos KV. Drug-induced tolerance: the effects of antibiotic pre-exposure in Stenotrophomonas maltophilia. Future Microbiol. 2020; 15(7):497–508. https://doi.org/10.2217/fmb-2019-0253 PMID: 32478618.

33. Yen P, Papin JA. History of antibiotic adaptation influences microbial evolutionary dynamics during subsequent treatment. PLoS Biol. 2017; 15(8):e2001586. https://doi.org/10.1371/journal.pbio.2001586 PMID: 28792497.

34. Blair JM, Webber MA, Baylay AJ, Ogbolu DO, Piddock LJ. Molecular mechanisms of antibiotic resistance. Nat Rev Microbiol. 2015; 13(1):42–51. https://doi.org/10.1038/nrmicro3380 PMID: 25435309.

35. Sanchez P, Alonso A, Martinez JL. Cloning and characterization of SmeT, a repressor of the Stenotrophomonas maltophilia multidrug efflux pump SmeDEF. Antimicrob Agents Chemother. 2002; 46 (11):3386–93. https://doi.org/10.1128/AAC.46.11.3386-3393.2002 PMID: 12384340.

36. Alonso A, Martinez JL. Cloning and characterization of SmeDEF, a novel multidrug efflux pump from Stenotrophomonas maltophilia. Antimicrob Agents Chemother. 2000; 44(11):3079–86. https://doi.org/10.1128/AAC.44.11.3079-3086.2000 PMID: 11036026.

37. Sanchez MB, Martinez JL. Overexpression of the efflux pumps SmeVWX and SmeDEF is a major cause of resistance to co-trimoxazole in Stenotrophomonas maltophilia. Antimicrob Agents Chemother. 2018;62(6). https://doi.org/http://doi.org/10.1128/AAC.00301-18 PMID: 29610195.

38. Noinaj N, Guillier M, Barnard TJ, Buchanan SK. TonB-dependent transporters: regulation, structure, and function. Annu Rev Microbiol. 2010; 64:43–60. https://doi.org/10.1146/annurev.micro.112408.134247 PMID: 20420522.

39. Calvopina K, Dulyayangkul P, Heesom KJ, Avison MB. TonB-dependent uptake of beta-lactam antibiotics in the opportunistic human pathogen Stenotrophomonas maltophilia. Mol Microbiol. 2020; 113 (2):492–503. https://doi.org/http://doi.org/10.1111/mmi.14434 PMID: 31773806.

40. Calvopina K, Dulyayangkul P, Avison MB. Mutations in ribosomal protein RplA or treatment with ribosomal acting antibiotics activates production of aminoglycoside efflux pump SmeYZ in Stenotrophomonas maltophilia. Antimicrob Agents Chemother. 2020;64(2). https://doi.org/10.1128/AAC.01524-19 PMID: 31712205.

41. Lin YT, Huang YW, Chen SJ, Chang CW, Yang TC. The SmeYZ efflux pump of Stenotrophomonas maltophilia contributes to drug resistance, virulence-related characteristics, and virulence in mice. Antimicrob Agents Chemother. 2015; 59(7):4067–73. https://doi.org/10.1128/AAC.00372-15 PMID: 25918140.

42. Wu CJ, Huang YW, Lin YT, Ning HC, Yang TC. Inactivation of SmeSyRy two-component regulatory system inversely regulates the expression of SmeYZ and SmeDEF efflux pumps in Stenotrophomonas maltophilia. PLoS One. 2016; 11(8):e0160943. https://doi.org/10.1371/journal.pone.0160943 PMID: 27513575.

43. Chittrakanwong J, Charoenlap N, Vanitshavit V, Sowatad A, Mongkolsuk S, Vattanaviboon P. The role of MfsR, a TetR-type transcriptional regulator, in adaptive protection of Stenotrophomonas maltophilia against benzalkonium chloride via the regulation of mfsQ. FEMS Microbiol Lett. 2021;368:fnab098. https://doi.org/10.1093/femsle/fnab098 PMID: 34329426.

44. Kalidasan V, Joseph N, Kumar S, Awang Hamat R, Neela VK. Iron and virulence in Stenotrophomonas maltophilia: All we know so far. Front Cell Infect Microbiol. 2018; 8:401. https://doi.org/10.3389/fcimb.2018.00401 PMID: 30483485.

45. Cunningham L, Gruer MJ, Guest JR. Transcriptional regulation of the aconitase genes (acnA and acnB) of Escherichia coli. Microbiology (Reading). 1997;143 (Pt 12):3795–805. https://doi.org/10.1099/00221287-143-12-3795 PMID: 9421904.

46. Tang Y, Guest JR. Direct evidence for mRNA binding and post-transcriptional regulation by Escherichia coli aconitases. Microbiology (Reading). 1999;145 (Pt 11):3069–79. https://doi.org/10.1099/00221287-145-11-3069 PMID: 10589714.

47. Kim HY, Go J, Lee KM, Oh YT, Yoon SS. Guanosine tetra- and pentaphosphate increase antibiotic tolerance by reducing reactive oxygen species production in Vibrio cholerae. J Biol Chem. 2018; 293 (15):5679–94. https://doi.org/10.1074/jbc.RA117.000383 PMID: 29475943.

48. Kohanski MA, Dwyer DJ, Hayete B, Lawrence CA, Collins JJ. A common mechanism of cellular death induced by bactericidal antibiotics. Cell. 2007; 130(5):797–810. https://doi.org/10.1016/j.cell.2007.06.049 PMID: 17803904.

49. Qian C-J, Li W, Li H., Ou D, Zhu-Ge Y-Y, Liu Y-D. Responses of genes for the uptake of glycine betaine in Virgibacillus halodenitrificans PDB-F2 under NaCl stress. Int Biodeterior Biodegrad. 2018; 132:192–9. https://doi.org/http://doi.org/10.1016/j.ibiod.2018.04.005

50. Reitzer LJ. Ammonia assimilation and the biosynthesis of glutamine, glutamate, aspartate, L-alanine, and D-alanine, p. 391–407. In F. C. Neidhardt, and R. Curtiss (ed.), Escherichia coli and Salmonella typhimurium, 2nd ed. ASM Press, Washington, D.C. 1996.

51. Zhao XL, Chen ZG, Yang TC, Jiang M, Wang J, Cheng ZX, et al. Glutamine promotes antibiotic uptake to kill multidrug-resistant uropathogenic bacteria. Sci Transl Med. 2021;13(625):eabj0716. https://doi.org/10.1126/scitranslmed.abj0716 PMID: 34936385.

52. Joo HK, Park YW, Jang YY, Lee JY. Structural analysis of glutamine synthetase from Helicobacter pylori. Sci Rep. 2018; 8(1):11657. https://doi.org/10.1038/s41598-018-30191-5 PMID: 30076387.

53. Amarasiri M, Sano D, Suzuki S. Understanding human health risks caused by antibiotic resistant bacteria (ARB) and antibiotic resistance genes (ARG) in water environments: Current knowledge and questions to be answered. Crit Rev Env Sci Tec. 2019; 50(19), 2016–59. https://doi.org/10.1080/10643389.2019.1692611
